# Diffusion tensor imaging in middle-aged headache sufferers in the general population: a cross-sectional population-based imaging study in the Nord-Trøndelag health study (HUNT-MRI)

**DOI:** 10.1186/s10194-019-1028-6

**Published:** 2019-07-10

**Authors:** Andreas Kattem Husøy, Live Eikenes, Asta K. Håberg, Knut Hagen, Lars Jacob Stovner

**Affiliations:** 1 Department of Neuromedicine and Movement Science, NTNU – Norwegian University of Science and Technology, 7491 Trondheim, Norway; 2 Norwegian Advisory Unit on Headaches, St. Olavs University Hospital, Trondheim, Norway; 3 Department of Circulation and Medical Imaging, NTNU – Norwegian University of Science and Technology, 7491 Trondheim, Norway

## Abstract

**Background:**

Several studies have investigated white matter with diffusion tensor imaging (DTI) in those suffering from headache, but so far only in clinic based samples and with conflicting results.

**Methods:**

In the present study, 1006 individuals (50–66 years) from the general population (Nord-Trøndelag Health Study) participated in an imaging study of the head at 1.5 T (HUNT-MRI). Hundred and ninety-six individuals were excluded because of errors in the data acquisition or brain pathology. Two hundred and forty-six of the remaining participants reported suffering from headache (69 from migraine and 76 from tension-type headache) the year prior to the scanning. DTI data were analysed with Tract-Based Spatial Statistics and automated tractography. Type of headache, frequency of attacks and evolution of headache were investigated for an association with white matter fractional anisotropy (FA), mean diffusivity (MD), axonal diffusivity (AD), radial diffusivity (RD) and tract volume. Correction for various demographical and clinical variables were performed.

**Results:**

Headache sufferers had widespread higher white matter MD, AD and RD compared to headache free individuals (*n* = 277). The effect sizes were mostly small with the largest seen in those with middle-age onset headache, who also had lower white matter FA. There were no associations between white matter microstructure and attack frequency or type of headache.

**Conclusion:**

Middle-age onset headache may be related to a widespread process in the white matter leading to altered microstructure.

**Electronic supplementary material:**

The online version of this article (10.1186/s10194-019-1028-6) contains supplementary material, which is available to authorized users.

## Background

Neuroimaging studies have demonstrated that primary headache disorders, e.g. migraine and tension-type headache (TTH), are not just paroxysmal conditions with no structural abnormalities of the brain. Ischemic strokes and white matter hyperintensities (WMH) have been reported to be more prevalent in those with migraine, in particular with aura, than in healthy controls [1, 2]. A previous study in the present population could not corroborate this, but found that those with TTH had more WMH than those without headache [3]. Quantitative brain measures have also been investigated. Several clinic-based studies have reported differences in cortical volume or thickness between those with and without headache, albeit not confirmed in the general population [4]. The relationship between headache and white matter (WM) microstructure has been examined with diffusion tensor imaging (DTI) but so far only in small clinic-based samples and with inconclusive results [5].

Several measures can be obtained from DTI that provide information on the white matter microstructure [6]. Fractional anisotropy (FA) is a scalar (range 0–1) that describes to which degree the diffusion is anisotropic. An FA value of 0 is present when the diffusion is isotropic (i.e. equal in all directions), which is the case for cerebrospinal fluid. An FA value of 1 on the other hand, is present when the diffusion occurs along one axis. Mean diffusivity (MD) is the total non-directional diffusion, axonal diffusivity (AD) is the diffusion along WM tracts and radial diffusivity (RD) is the diffusion perpendicular to AD, i.e. across WM tracts.

Three previous studies reported no differences in DTI indices between migraine patients and controls using Tract-Based Spatial Statistics (TBSS) [7, 8] or a region-of-interest approach (in the internal capsule and subcortical WM) [9]. The participants were in early adulthood or middle-aged, and WMH were corrected for. Similar, Liu et al. [10] followed 36 individuals newly diagnosed with migraine without aura and found no changes in WM FA, MD, AD or RD after 1 year.

Several other studies have found differences in WM microstructure between headache patients and controls. Three studies reported higher MD or RD in migraine patients compared to controls in early adulthood using TBSS [11] or DTI tract-average values [12, 13]. In contrast, other studies using TBSS reported migraineurs in their teens or early adulthood to have lower MD or RD in several WM regions compared to controls [14–17] and one study [14] also reported decreased AD in several WM regions in migraineurs. In contrast to the conflicting MD, AD and RD results, lower FA has consistently been reported in migraine patients [11, 13, 16–20]. No study has investigated the relationship between WM microstructure and TTH.

Common to all previous studies is the use of clinical samples making them vulnerable to selection bias. This may explain the conflicting results. Furthermore, the samples were relatively small (typically 20 cases and 20 controls) increasing the likelihood of the samples not being representative and rendering them unable to detect potentially small but actual differences [21]. Most of the studies reporting differences in WM microstructure did not correct their analyses for WMH [11, 14, 15, 18–20, 22, 23], which is known to influence DTI measures [24].

The present study aimed to investigate WM microstructure in those suffering from headache in a large sample from the general population where correction for several demographical and clinical variables were available. Type of headache (migraine or TTH), frequency of headache attacks, and evolution of headache complaints were investigated for a relationship with regional WM microstructure with TBSS. To examine if potential regional differences in white matter microstructure could be reflected at the level of WM tracts, tract volumes and tract-average values of DTI indices were obtained with automated tractography and compared between groups.

## Methods

### Cohort

The Nord-Trøndelag Health Study (Norwegian acronym HUNT) is an on-going large population-based study across several waves: 1984–1986 (HUNT1), 1995–1997 (HUNT2), 2006–2008 (HUNT3) and presently (HUNT4) in the county of Trøndelag (formerly Nord-Trøndelag), Norway. In a neuroimaging sub-study of HUNT3 (HUNT-MRI), 1006 individuals (530 women) between 50 and 66 years who had previously participated in HUNT1, HUNT2 and HUNT3, and lived within 45 min of the location of the scanning, underwent brain imaging with a standardized MRI protocol. Exclusion criteria were restricted to standard safety contraindication to MRI, i.e. pacemaker, severe claustrophobia or body weight above 150 kg. The scanning took place between the 21st of July 2007 and the 10th of December 2009. The mean time from answering the questionnaire in HUNT3 and being scanned was 1.2 years. Details about the imaging procedure and the recruitment of participants to the HUNT-MRI study have been published previously [25]. Compared to the general population, the participants of the HUNT-MRI study had possibly somewhat reduced risk of cardiovascular disease [25].

### Headache diagnoses

Based on their answers (“yes/no”) to the initial screening question of a headache questionnaire (“have you suffered from headache during the last 12 months?”), HUNT3 participants were classified as either headache sufferers or headache nonsufferers. Suffering from any headache had a sensitivity of 88% and a specificity of 86% [26]. Headache sufferers were further asked to answer 13 headache questions designed to determine whether the person suffered from migraine or TTH. The migraine and TTH diagnoses were based on the criteria of the 2nd edition of the International Classification of Headache Disorders (ICHD-II). However, since the infrequent TTH diagnosis had a low specificity, only those with headache ≥1 day per month were given the TTH diagnosis [26]. For migraine, the sensitivity was 51% and the specificity was 95% and for TTH the sensitivity was 96% and the specificity was 69% [26]. Headache sufferers not fulfilling the criteria of either migraine or TTH were categorized as having unclassified headache. In the present study no analyses were performed on this group separately. Headache sufferers in HUNT3 were also categorized into four separate groups based on the frequency of headache attacks (< 1 day/month; 1–6 days/month; 7–14 days days/month; > 14 days/month).

Information on evolution of headache status was based on identical headache screening questions in HUNT2 and HUNT3. Participants who had answered both questionnaires were categorized into four mutually exclusive categories: previous headache (headache in HUNT2 but not in HUNT3), new onset headache (no headache in HUNT2 but headache in HUNT3), persistent headache (headache in both HUNT2 and HUNT3) and headache free (headache in neither HUNT2 nor HUNT3).

### Demographical and clinical variables

As part of the HUNT surveys demographics and health related data were collected with questionnaires and various supplementary investigations. In the present study a selection of variables was used to highlight similarities and differences between the various headache groups and the headache free and between the four headache attack frequency groups. Information on age (continuous), sex (dichotomous), body mass index (BMI, continuous), blood pressure (continuous), non-fasting glucose (continuous), cholesterol (continuous), smoking (dichotomous), Hospital Anxiety and Depression Scale (HADS, continuous), chronic pain (body pain for more than 6 months, dichotomous) and consumption of alcohol (ordinal from 1 [never] to 8 [4–7 times/week] but dichotomized at ≥1/week for these analyses) and over-the-counter painkillers (dichotomous) was collected from the HUNT3 survey. Information on education (dichotomous) was collected from the HUNT2 survey. Differences in age were evaluated with independent t-tests and differences in sex were evaluated with χ^2^ tests. Analysis of Covariance or binary logistic regression, corrected for age and sex, was used to investigate for differences in the other variables. The analyses were carried out in SPSS version 25 and thresholded at *P* < 0.05 (two-tailed).

### MRI scanning

All MRI examinations were performed on the same 1.5 T General Electric Signa HDx scanner equipped with an eight-channel head coil and software version pre-14.0 M (GE Healthcare). All participants underwent the same scan protocol and no scanner updates were performed during the HUNT-MRI study. In the present study, data from the T1-weighted volume, transverse T2, FLAIR and DTI sequences, were used. Scan parameters for the T1, T2 and FLAIR sequences are listed in Additional file 1: Table S1. The DTI sequence was a single-shot balanced-echo EPI acquired in 40 non-colinear directions with b = 1000 s/mm^3^ and 5 b = 0 images using the following parameters: TR = 13,500 ms, TE = 104 ms, FOV 240 × 240 mm, slice thickness 2.5 mm, acquisitions matrix 96 × 96. The images were automatically zero-padded in k-space from 96 × 96 to 256 × 256 and reconstructed giving a resolution of 0.9275 × 0.9275 × 2.5 mm^3^. 60 transversal slices with no gaps were acquired giving full brain coverage.

### White matter hyperintensities and intracranial volume

Using the FLAIR images, the load of hemispheric WMH was evaluated by an experienced neuroradiologist, blinded to headache status, using the Fazekas scale (0–3) [3]. Intracranial volume (ICV) was estimated in Statistical Parametric Mapping 8 with an automated version of the reverse brain mask method using the T1 and T2 images [27].

### DTI analysis

DTI analysis was performed with two methods: TBSS (FMRIB Software Library (FSL), The Oxford Centre for functional MRI of the Brain (FMRIB), Oxford, UK; www.fmrib.ox.ac.uk/fsl) and an automated tractography method [28]. Common to both methods, image artefacts due to motion and current distortions were minimized by registration of the DTI acquisition to the b = 0 image using affine registration.

### TBSS analyses

The brain was extracted using the Brain Extraction Tool (BET, part of FSL). The FMRIBs Diffusion Toolbox (FDT) was used to fit a diffusion tensor to the raw diffusion data. Voxel-wise maps of the FA, MD, AD and RD were calculated for the headache and control groups. Voxel-wise statistical analysis of the diffusion data was performed using TBSS [29, 30]. Briefly, all subjects’ FA data were aligned into a common space using the nonlinear registration tool FNIRT [31, 32] (which uses a b-spline representation of the registration warp field [33]). A mean FA image was created from all the FA images and thinned to create a skeletonized mean FA representing the centers of all tracts common to all the subjects in the analysis. The mean FA skeleton was thresholded to FA ≥ 0.2 to include major WM tracts but exclude peripheral tracts and grey matter. Each subject’s aligned FA data were then projected onto this skeleton. The skeletonization process was also applied to MD, AD and RD, and the statistical comparisons of these data were then restricted to voxels in the WM skeleton. The resulting skeletonized data were consequently fed into voxel-wise cross-subject statistics in Randomise as described below.

### Automated tractography

The automated tractography segmentation procedure applied in the present study have been described previously [34]. Briefly, q-ball reconstruction was used to parameterize voxel diffusion profiles, and up to three principal diffusion directions were determined for each voxel [35]. The Camino package was used to generate streamlines using the interpolated deterministic streamlining method, with an FA threshold of 0.15. All voxels with an FA value > 0.25 were used as seed values.

The mean b = 0 volumes were registered to the MNI152 template using FLIRT. A custom group template was created by averaging the registered volumes. The b = 0 volumes were then nonlinearly registered to the template with FNIRT and the deformation fields produced by FNIRT was used to warp the streamlines from each subject to the group template.

To find consistent bundles of streamlines across subjects an approach previously described by Visser et al. was used [28]. Before clustering, all streamlines were linearly resampled to 25 points, and the streamlines from all subjects were concatenated. Clustering was performed on the merged data set consisting of streamlines from all subjects. The multisubject data set was randomly partitioned into subsets of 10,000 streamlines, and in each of these subsets 250 clusters were identified by using hierarchical clustering. The clustering step was repeated 100 times with different random partitions to obtain a stable segmentation by selecting the cluster assignments that occurred most often for each streamline to find statistics indicating the consistency of these assignments between repetitions. Based on anatomical knowledge, WM tracts were identified in 10 randomly selected individuals by manually assigning the sets of labels (from the 250 labels) that corresponded to the following WM tracts: corpus callosum (CC), cingulate (CING), corticospinal tract (CST), inferior fronto-occipital fasciculus (IFOF), inferior longitudinal fasciculus (ILF), optic radiation (OR), superior longitudinal fasciculus (SLF), and uncinate fasciculus. For each subject, the clusters were extracted with pruning (thresholding). Regions of interests (ROIs) were made for the fiber tracts and converted into subject diffusion space to extract mean FA, MD, AD and RD for each tract. Tract volume was calculated for each WM tract by adding the number of voxels containing at least one streamline and multiplying by voxel volume. It is important to note that this value reflects the number of voxels within the tract that exceeded the tracking FA threshold and might deviate from the actual volume. The diffusivity indices and volumes for the tracts in the left and right hemispheres were merged and imported into SPSS version 25.

### Statistical approach to DTI measures

In the present study, several group comparisons were performed. First, those suffering from any headache (in HUNT3) were compared to the headache free group. Second, the following five headache subcategories were compared to the headache free group: migraine, TTH, previous headache, new onset headache and persistent headache. Third, those with migraine were compared to those with TTH. Fourth, correlation analyses between the frequency of headache attacks and the DTI indices were performed. All these analyses were initially corrected for age and sex (and ICV in the volume analyses) (Model 1) but rerun three times to correct for variables that were thought to possibly affect the results. First, since WMH affects the integrity of WM [24], the Fazekas score was added as a covariate (Model 2). Second, the Fazekas score was excluded as a covariate and clinical variables that were found to be significantly different between the headache sufferers and the headache free (HADS score, presence of chronic pain and consumption of alcohol [the ordinal variable] and over-the-counter painkillers) were added (Model 3). Third, both the Fazekas score and the clinical variables were added as covariates (Model 4).

The Randomise tool in FSL was used to conduct permutation-based non-parametric tests to investigate spatial differences in DTI indices in WM obtained with TBSS [36]. Threshold-free cluster enhancement (TFCE), corrected for multiple comparisons with family-wise error rate (FWE) and thresholded at *P* < 0.05 (two-tailed), was used to investigate group differences in WM FA, MD, AD and RD. Tract-average values and tract-volumes obtained with automated tractography were analysed with ANCOVA in SPSS version 25. *P*-values were considered significant at a 0.05 level (two-tailed). In total 1280 comparisons were performed in the automated tractography analyses. The volume analyses were corrected for ICV. Effect sizes (Cohen’s d) were calculated from the TBSS and automated tractography analyses.

## Results

### Basic characteristics of the present population

Of the 1006 MRI examinations, 97 had missing DTI (due to empty folders, no DTI acquisition or abrupted DTI acquisition) and 32 were excluded due to DTI artefacts (ghosting, signal void or deformation). Of the 877 successful DTI scans, 2 individuals with multiple sclerosis were excluded and another 65 individuals were excluded due to pathology [37]. Some of the included individuals had minor intracranial abnormalities (Additional file 2: Table S2 and Additional file 3: Table S3). In total, 810 participants had successful DTI scans and were eligible for inclusion in the present study. However, since some of these participants did not fulfil the criteria for inclusion in one of the headache categories because of lacking data in either HUNT2 or HUNT3, the total number of included participants was 640.

Tables 1 and 2 shows the basic characteristics of the present headache groups. Except for the new onset headache group, all headache groups had a significantly higher percentage of women than the headache free group. The any headache, migraine and persistent headache groups also included individuals that were significantly younger and had a lower consumption of alcohol than the headache free group. Except for the previous and new onset headache groups, all headache groups had a higher HADS score than the headache free group. Headache sufferers used significantly more over-the-counter painkillers than the headache free, whereas no significant differences were found with regard to BMI, blood pressure, non-fasting glucose, cholesterol, daily smoking or level of education. HADS scores, the presence of chronic pain and consumption of over-the-counter painkillers showed a significant positive association with frequency of headache attacks.

**Table 1:** Basic characteristics of participants in the present study

Variables	Headache status						
	Any headache in HUNT3	Migraine in HUNT3	TTH in HUNT3	Previous headache	New onset headache	Persistent headache	Headache free
	*n* = 246	*n* = 69	*n* = 76	*n* = 117	*n* = 49	*n* = 178	n = 277
Demographics							
Women (n [%] {missing})^1^	153*** [62.2] {0}	53*** [76.8] {0}	42* [55.3] {0}	70*** [59.8] {0}	25 [51.0] {0}	117*** [65.7] {0}	109 [39.4] {0}
Age (mean [SD] {missing})^2^	58.0* [4.1] {0}	57.5* [4.3] {0}	57.9 [4.0] {0}	58.5 [4.1] {0}	58.6 [4.6] {0}	57.7** [4.1] {0}	58.7 [4.1] {0}
Education > 12 years (n [%] {missing})^3^	75 [30.5] {0}	22 [31.9] {0}	25 [32.9] {0}	41 [35.0] {0}	13 [26.5] {0}	59 [33.1] {0}	102 [36.8] {0}
Health-related							
BMI (mean [SD] {missing})^4^	26.7 [4.0] {1}	26.5 [4.2] {0}	27.0 [4.3] {0}	26.8 [3.8] {0}	27.1 [3.8] {1}	26.4 [4.0] {0}	26.9 [3.5] {0}
SBP (mean [SD] {missing})^4^	132.2 [18.3] {4}	132.1 [18.8] {1}	132.4 [18.0] {1}	131.3 [17.5] {0}	136.1 [19.5] {1}	131.3 [18.4] {3}	130.1 [15.7] {1}
DBP (mean [SD] {missing}) ^4^	76.7 [11.8] {3}	75.4 [12.1] {1}	77.4 [11.2] {0}	74.5 [10.3 ] {0}	78.3 [12.7] {1}	75.9 [11.8] {2}	76.1 [10.3] {1}
Non-fasting glucose (mean [SD] {missing})^4^	5.5 [1.2] {6}	5.3 [0.6] {3}	5.6 [1.4] {2}	5.7 [1.4] {12}	5.6 [1.4] {0}	5.4 [1.1] {4}	5.6 [1.8] {13}
Cholesterol (mean [SD] {missing})^4^	5.8 [1.0] {6}	5.8 [1.2] {3}	5.8 [1.0] {2}	5.7 [1.0] {12}	5.8 [0.9] {0}	5.8 [1.1] {4}	5.7 [1.0] {13}
Daily smoking (n [%] {missing})^3^	38 [15.4] {0}	15 [21.7] {0}	11 [14.5] {0}	16 [13.7] {0}	7 [14.3] {0}	29 [16.3] {0}	41 [14.8] {0}
HADS total score (mean [SD] {missing})^4^	7.8*** [5.9] {1}	8.0** [5.8] {0}	7.7** [5.9] {0}	5.9 [4.5] {0}	7.3 [5.6] {0}	8.2*** [6.1] {1}	6.0 [4.9] {3}
Chronic pain last 6 months (n [%] {missing})^3^	137*** [56.7] {3}	38** [55.1] {1}	46*** [60.5] {0}	49 [41.9] {3}	25* [51] {0}	105*** [59.0] {3}	91 [32.9] {4}
Consuming alcohol ≥1/week (n [%] {missing})^3^	108** [43.9] {4}	27** [39.1] {2}	43 [56.6] {0}	67 [57.3] {1}	25 [51.0] {2}	73*** [41.0] {2}	163 [58.8] {0}
Painkillers ≥1/week (n [%] {missing})^3^	170*** [69.1] {4}	53*** [76.8] {0}	50*** [65.8] {1}	53*** [45.3] {2}	30*** [61.2] {0}	130*** [73.0] {4}	70 [25.3] {5}

*P < 0.05 (compared to headache free)

**P < 0.01 (compared to headache free)

***P < 0.001 (compared to headache free)

^1^Chi-square test; ^2^ Independent t-test; ^3^ Binary logistic regression; ^4^ Analysis of Co-Variance

*n* Number of individuals, *SD* Standard Deviation, *SBP* Systolic Blood Pressure, *DBP* Diastolic Blood Pressure, *HADS* Hospital Anxiety and Depression Scale

**Table 2:** Basic characteristics of the present headache sufferers based on the frequency of headache attacks

Variables	Frequency of headache attacks†			
	Headache < 1 day/month	Headache 1–6 days/month	Headache 7–14 days/month	Headache > 14 days/month
	n = 69	*n* = 135	*n* = 28	n = 13
Demographics				
Women (n [%] {missing})^1^	40 [58.0] {0}	84 [62.2] {0}	21 [75.0] {0}	7 [53.8] {0}
Age (mean [SD] {missing})^2^	58.0 [4.6] {0}	58.4 [4.0] {0}	58.8 [3.6] {0}	61.2 [3.6] {0}
Education > 12 years (n [%] {missing})^3^	20 [30.0] {0}	44 [32.6] {0}	9 [32.1] {0}	2 [15.4] {0}
Health-related				
BMI (mean [SD] {missing})^4^	26.3 [4.0] {1}	26.8 [4.0] {0}	27.2 [3.9] {0}	27.1 [3.8] {0}
SBP (mean [SD] {missing}) ^4^	133.2 [17.0] {2}	131.4 [18.6] {2}	131.5 [18.6] {0}	135.1 [21.3] {0}
DBP (mean [SD] {missing}) ^4^	78.1 [11.0] {2}	76.4 [11.5] {1}	74.5 [13.1] {0}	77.7 [15.5] {0}
Non-fasting glucose (mean [SD] {missing})	5.4 [0.9] {1}	5.4 [1.2] {4}	5.4 [0.8] {1}	5.9 [2.9] {1}
Cholesterol (mean [SD] {missing})	5.6 [0.9] {1}	5.9 [1.1] {4}	5.6 [0.9] {1}	5.7 [1.2] {1}
Daily smoking (n [%] {missing})^3^	9 [13.0] {0}	26 [19.3] {0}	3 [10.7] {0}	0 [0] {0}
HADS total score (mean [SD] {missing})^4^**	6.1 [4.7] {0}	8.1 [6.0] {1}	10.7 [7.0] {0}	10.0 [5.7] {0}
Chronic pain last 6 months (n [%] {missing})^3^*	32 [46.4] {1}	73 [54.1] {1}	21 [75.0] {0}	10 [76.9] {1}
Consuming alcohol ≥1/week (n [%] {missing})^3^	29 [42.0] {2}	63 [46.7] {2}	12 [42.9] {0}	3 [23.1] {0}
Painkillers ≥1/week (n [%] {missing})^3^*	40 [58.0] {0}	95 [70.4] {4}	25 [89.3] {0}	9 [69.2] {0}

*P < 0.05

**P < 0.01

†One headache sufferer lacked data on attack frequency

^1^ Chi-square test; ^2^ Analysis of Variance; ^3^ Binary logistic regression; ^4^ Analysis of Co-Variance

*n* Number of individuals, *SD* Standard Deviation, *SBP* Systolic Blood Pressure, *DBP* Diastolic Blood Pressure, *HADS* Hospital Anxiety and Depression Scale

The WMH load was similar in the different headache groups and the headache free group and was not correlated with the frequency of headache attacks (Additional file 4: Table S4). The frequency of headache attacks was quite similar across the headache groups. Still, headache < 1 day/month seemed to be slightly more common for those with new onset headache. Note that owing to the diagnostic criteria, none of those with TTH had headache < 1 day/month (Additional file 5: Table S5).

### TBSS analyses corrected for age and sex (model 1, Fig. 1)

The any headache group had significantly higher MD, AD and RD compared to the headache free group. The higher MD and AD were present in all major WM tracts, whereas higher RD was present in most major WM tracts. Those with migraine or TTH had higher AD than headache free in several areas of the TBSS skeleton, most prominent in CC, CST, IFOF, ILF and left SLF. Individuals with previous headache had widespread higher MD and AD in all major WM tracts compared to the headache free, whereas those with persistent headache had higher AD than the headache free, mostly in the left hemisphere in CST, IFOF, ILF and SLF. Those suffering from new onset headache had significantly higher MD, AD and RD and significantly reduced FA in all major WM tracts compared to the headache free. There were no differences in any of the DTI indices between migraine and TTH and no correlation between any of the DTI indices and frequency of headache attacks.

**Fig. 1:**
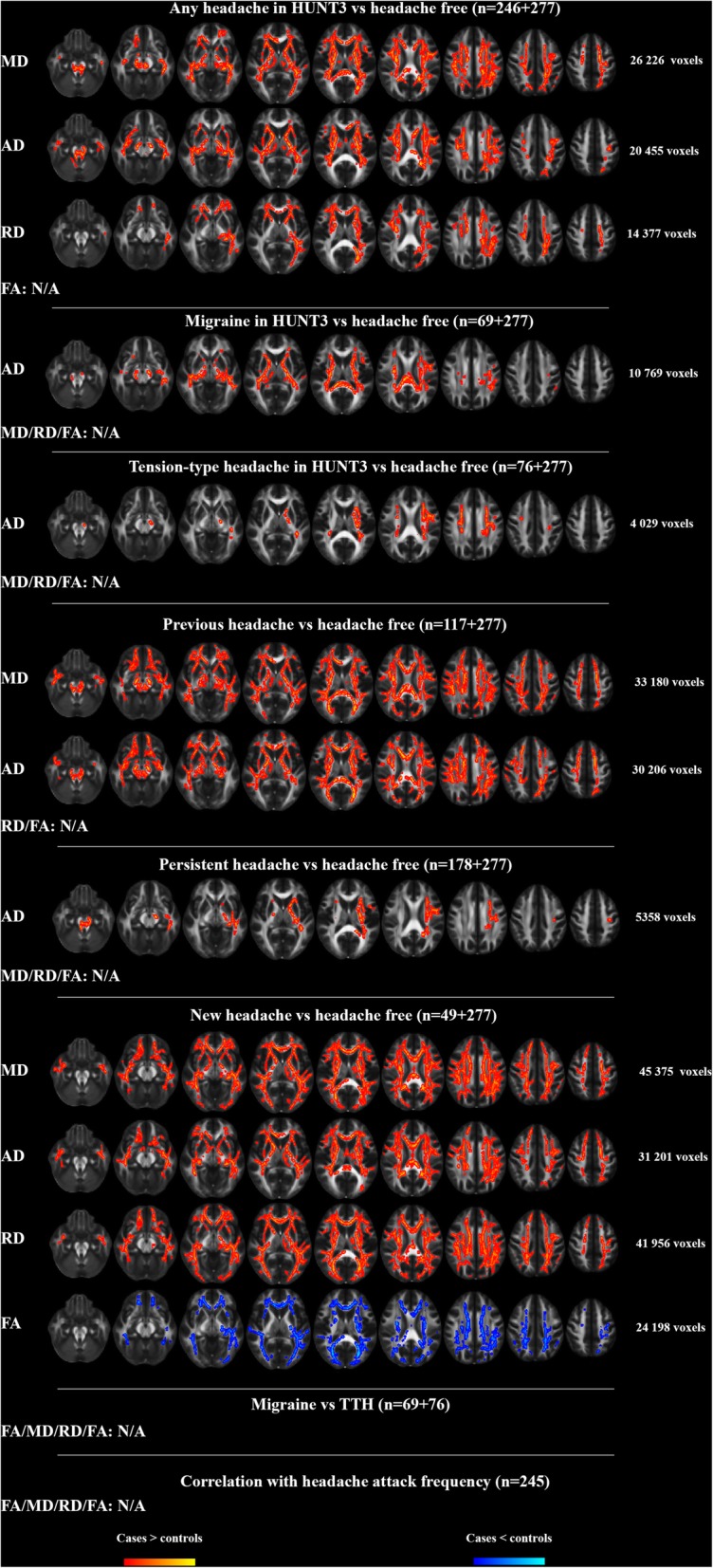
Between group differences in white matter FA, MD, AD and RD in the TBSS analyses. Significance level was *P* < 0.05 (two-tailed) corrected for age and sex and multiple comparisons with Threshold Free Cluster Enhancement and Family-Wise Error rate as implemented in Randomise. To improve visualization, the group differences were “thickened” using the tbss_fill script in FSL. The FSL 1 mm mean FA template was used as background image

### TBSS analyses corrected for age, sex and WMH (model 2, Fig. 2)

Adding the Fazekas score as a covariate decreased the number of voxels with higher MD and AD in the any headache group compared to the headache free group, and the RD differences became insignificant. Similarly, the extent of higher AD in migraineurs compared to the headache free decreased (now present mostly in CST) and higher AD in those with TTH was present in a small area in SLF. Correction for WMH had negligible impact on the comparisons between those with previous headache and the headache free but rendered the differences in AD between those with persistent headache and the headache free insignificant. The extent of differences in FA, MD, AD and RD between those with new onset headache and the headache free was slightly reduced but still present in all major WM tracts. As in statistical Model 1, there were no differences in any of the DTI indices between migraine and TTH and no correlation between any of the DTI indices and frequency of headache attacks.

**Fig. 2:**
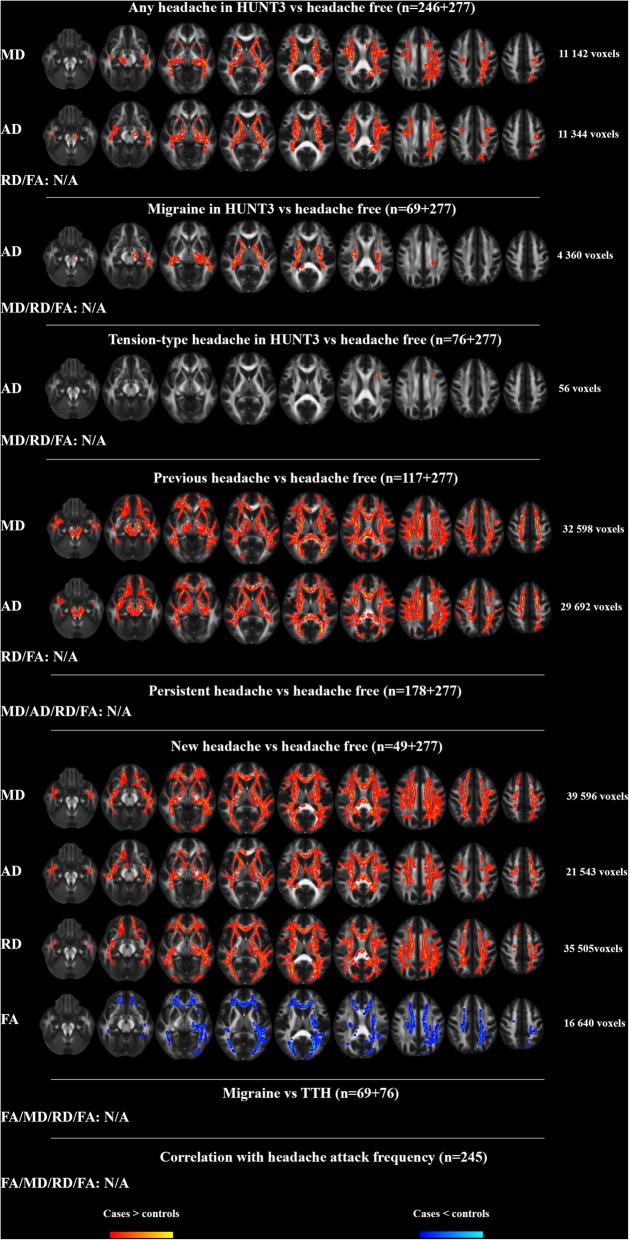
WMH-corrected differences in white matter FA, MD, AD and RD in the TBSS analyses. In addition, there was correction for age, sex and multiple comparisons with Threshold Free Cluster Enhancement and Family-Wise Error rate as implemented in Randomise. Significance level was *P* < 0.05 (two-tailed). To improve visualization, the group differences were “thickened” using the tbss_fill script in FSL. The FSL 1 mm mean FA template was used as background image

### TBSS analyses corrected for age, sex, HADS score, chronic pain and consumption of alcohol and over-the-counter painkillers (model 3, Additional file 7: Figure S1)

When correcting for age, sex, and the four clinical variables, those suffering from any headache still had higher MD and AD compared to the headache free. The extent of the MD differences was decreased and included CST, IFOF, ILF and SLF in the left hemisphere. The differences in AD were still present in virtually all WM tracts. For migraine, higher AD was present in the same WM tracts as when correcting only for age and sex, and higher MD emerged in an area of the right CST. Those with TTH had higher AD than the headache free in left CST and left SLF. The number of voxels with higher AD in those with previous headache compared to the headache free were markedly reduced, whereas the differences in MD were barely affected. Furthermore, some scattered areas with significantly higher RD in those with previous headache appeared. There were no differences in any of the DTI indices between those with persistent headache and the headache free. The new onset headache group had higher MD, AD and RD compared to the headache free group. The number of voxels that were significantly different between the two groups decreased but was still present in all WM tracts. No significant differences in FA were found. There were no differences in any of the DTI indices between migraine and TTH and no correlation between any of the DTI indices and frequency of headache attacks (as in Model 1 and 2).

### TBSS analyses corrected for age, sex, HADS score, chronic pain, consumption of alcohol and over-the-counter painkillers and WMH (model 4, Additional file 8: Figure S2)

Including all covariates in the statistical model made the difference in MD between the any headache group and the headache free group insignificant and markedly reduced the extent of higher AD to mostly include left CST and left SLF. Although considerably reduced, the higher AD in migraineurs compared to the headache free was still present in CC, right IFOF, right ILF and right CST. No differences were found between those with TTH and the headache free. Those with previous headache had higher MD in all areas of the TBSS skeleton and some areas of higher AD and RD in virtually all major WM tracts compared to the headache free. There were no differences in any of the DTI indices between those with persistent headache and the headache free. The new onset headache group had considerably higher MD, AD and RD compared to the headache free group in virtually all major WM tracts. There were no differences in any of the DTI indices between migraine and TTH and no correlation between any of the DTI indices and frequency of headache attacks (as in Model 1, 2 and 3).

### Effect sizes in the TBSS analyses

Table 3 shows the mean and peak (absolute numbers) Cohen’s d values of the TBSS analyses (except for the correlation analyses on attack frequency) corrected for age and sex as well as the mean Cohen’s d values of only the significant voxels in each comparison. The mean effect sizes were very small to small (range = 0.07–0.17) and relatively similar across the different comparisons. The peak Cohen’s d values were mostly medium to large (range = 0.12–0.88) with the largest values present in the comparisons between the new onset headache and the headache free groups and in the direct comparison of migraine and TTH. Considering only the voxels that were significant in each comparison, the mean Cohen’s d values were small (range = 0.15–0.29).

**Table 3:** Mean Cohen’s d values of the TBSS analyses corrected for age and sex. In parenthesis are peak Cohen’s d values and in brackets are mean Cohen’s d values when considering only the significant voxels

	FA	MD	AD	RD
Any headache in HUNT3 vs headache free	0.07 (0.41)	0.08 (0.46) [0.15]	0.08 (0.42) [0.15]	0.08 (0.41) [0.16]
Migraine in HUNT3 vs headache free	0.04 (0.36)	0.11 (0.58)	0.11 (0.63) [0.23]	0.11 (0.58)
TTH in HUNT3 vs headache free	0.10 (0.12)	0.11 (0.63)	0.11 (0.62) [0.29]	0.11 (0.63)
Previous headache vs headache free	0.09 (0.45)	0.10 (0.52) [0.18]	0.10 (0.53) [0.17]	0.09 (0.53)
Persistent headache vs headache free	0.08 (0.44)	0.08 (0.43)	0.08 (0.48) [0.18]	0.08 (0.40)
New onset headache vs headache free	0.14 (0.77) [0.26]	0.17 (0.88) [0.27]	0.14 (0.83) [0.25]	0.16 (0.86) [0.27]
Migraine vs TTH	0.14 (0.32)	0.13 (0.71)	0.14 (0.82)	0.13 (0.71)

### Tract-average DTI indices

Additional file 6: Table S6 shows the results of the comparisons of WM tract average DTI indices between the headache groups and the headache free group obtained with automated tractography (only significant differences are shown). When correcting for age and sex (Model 1) the results showed that those suffering from headache had higher MD and AD in ILF compared to the headache free. Higher MD and AD were particularly notable in those with TTH or new onset headache. In addition, those with previous headache had higher AD in CST compared to the headache free.

Adding the Fazekas score as a covariate (Model 2) resulted in fewer significant results and had in particular an impact on IFOF when comparing TTH with the headache free. In addition, those with persistent headache had lower RD of the CING compared to the headache free. The difference in FA of the CING between those with persistent headache and the headache free was not affected by correction for WMH.

When age, sex, HADS score, chronic pain and consumption of alcohol and over-the-counter painkillers were included as covariates (Model 3) almost all previous significant results were eliminated. In contrast, those with migraine and previous headache now had lower FA of the CST compared to the headache free.

When all covariates were included in the statistical model (Model 4) only one comparison showed significance, i.e. those with previous headache had lower FA in CST compared to the headache free. As the Cohen’s d values in Additional file 6: Table S6 shows, there were relatively small differences in tract-average DTI indices between the headache groups and the headache free group (mean = 0.25, median = 0.25, std. deviation = 0.10 and range [0.03–0.41]).

### Tract volumes

Table 4 summarizes the results of the comparisons of tract volumes between the headache groups and the headache free group obtained with automated tractography (only significant differences are shown). Those with new onset headache had lower volume of the CC and the IFOF than the headache free in all statistical models. Similar, those with TTH had a lower volume of the CC than the headache free in all statistical models. Individuals with previous headache had lower volume of the IFOF compared to the headache free in Model 3 and Model 4. The Cohen’s d values in Table 5 shows that there were relatively small differences in tract volumes between the headache groups and the headache free group (mean = 0.26, median = 0.27, std. deviation = 0.10 and range [0.07–0.39]).

**Table 4:** Volumes (mean values with standard deviations in parenthesis) of white matter tracts with significant differences between various headache groups obtained via automated tractography. Only significant comparisons are shown

White matter tract	Diffusivity index	Headache status		Cohen’s d	*P*-values
		Headache free	TTH in HUNT3		
CC	Model 1	133,199.55 (27,111.58)	128,233.64 (26,728.43)	0.18	0.015
	Model 2	133,199.55 (27,111.58)	128,233.64 (26,728.43)	0.18	0.027
	Model 3	133,333.22 (27,393.97)	128,279.36 (26,905.43)	0.19	0.013
	Model 4	133,333.22 (27,393.97)	128,279.36 (26,905.43)	0.19	0.021
		Headache free	Previous headache		
IFOF	Model 3	55,877.84 (12,506.42)	52,031.05 (11,029.22)	0.33	0.044
	Model 4	55,877.84 (12,506.42)	52,031.05 (11,029.22)	0.33	0.044
		Headache free	New onset headache		
CC	Model 1	133,199.55 (27,111.58)	123,362.10 (23,524.54)	0.39	0.001
	Model 2	133,199.55 (27,111.58)	123,362.10 (23,524.54)	0.39	0.002
	Model 3	133,333.22 (27,393.97)	123,476.03 (24,022.37)	0.38	0.003
	Model 4	133,333.22 (27,393.97)	123,476.03 (24,022.37)	0.38	0.004
IFOF	Model 1	55,849.15 (12,415.01)	52,573.81 (12,236.59)	0.27	0.013
	Model 2	55,849.15 (12,415.01)	52,573.81 (12,236.59)	0.27	0.017
	Model 3	55,877.84 (12,506.42)	52,797.27 (12,271.63)	0.25	0.014
	Model 4	55,877.84 (12,506.42)	52,797.27 (12,271.63)	0.25	0.017

Model 1 = Corrected for age, sex and ICV

Model 2 = Corrected for age, sex, ICV and WMH

Model 3 = Corrected for age, sex, ICV, HADS, chronic pain and consumption of alcohol and over-the-counter painkillers

Model 4 = Corrected for age, sex, ICV, WMH, HADS, chronic pain and consumption of alcohol and over-the-counter painkillers

## Discussion

The present TBSS analyses showed that those suffering from headache had widespread higher WM MD, AD and RD compared to headache free individuals. The largest effects were seen in those with new onset headache, who also had a decrease in WM FA compared to the headache free in some of the statistical models. Interestingly, the WM microstructure of individuals with persistent headache was quite similar to the WM microstructure of the headache free and there were no correlations between frequency of headache attacks and DTI indices. No significant difference in WM microstructure between migraine and TTH was detected. Several analyses with different covariates were performed and the relevance of these will be further discussed.

The higher WM AD in those with TTH and/or persistent headache compared to the headache free could be explained by WMH. For migraine on the other hand, a combination of WMH, HADS score, chronic pain and the consumption of alcohol and over-the-counter painkillers almost completely explained the higher WM AD. In contrast, the differences in WM microstructure between those with previous or new onset headache and the headache free were to a far lesser degree explained by these variables. Correction for WMH had virtually no impact on the WM microstructure of those with previous headache and only slightly decreased the difference in WM microstructure between the new onset headache group and the headache free. Correcting for HADS, chronic pain, alcohol and over-the-counter painkillers made the FA comparison between the new onset headache group and the headache free insignificant, but did not eliminate the significant differences in MD, AD and RD.

It is of interest that the differences in WM microstructure between those with and without headache were widespread and not confined to certain WM tracts. This suggests a widespread process in the white matter leading to altered microstructure. Furthermore, this putative process led to higher diffusivity both parallel (AD) and perpendicular (RD) to the axons. According to the literature, AD is positively associated with axonal integrity and RD negatively associated with myelination [38]. AD and RD (and thus MD) seem to be more sensitive measures of WM microstructure with regard to headache status than FA. Presumably, a high degree of overlap of location of voxels with higher AD and RD led to minimal change in FA.

It is hard to find a pathophysiological plausible explanation for the fact that those with previous or new onset headache had a microstructure of the WM far more different to the headache free’s than those with persistent headache had. The presence of intracranial abnormalities did probably not explain the results as they were uncommon since most participants with intracranial abnormalities were excluded from the DTI analysis, and in the remaining sample the frequency and type of findings were quite similar across the different headache groups. Interestingly, those with previous or new onset headache were more similar to the headache free in terms of demographical and clinical characteristics than those with persistent headache. One might hypothesize that for instance a high consumption of painkillers or a low consumption of alcohol, both of which were present in those with persistent headache, protect the brain from damage caused by headache. Since old ischemic lesions lead to increased MD and decreased FA, and several painkillers share characteristics with antiplatelet drugs used to prevent ischemic events, the usage of painkillers could conceivably affect the microstructure of the white matter [6, 39]. Alcohol has previously been shown to increase MD and decrease FA [40]. However, correction for these variables showed that the present level of alcohol and painkiller consumption could not explain the results.

Primary headaches are usually developed in the teens or early adulthood whereas secondary headaches more often start later in life [41, 42]. Considering that the participants in the present study were aged 50–66 years this may be of importance. Since individuals reporting new onset headache in HUNT3 actually had a middle-age onset headache, one may speculate that some of them suffered from some sort of secondary headache, and furthermore, that the widespread higher WM diffusion in these individuals is reflective of a brain process leading to headache.

The differences in WM microstructure between those with and without headache were not associated with type of headache. Both migraine and TTH had a slightly higher WM AD when compared to the headache free. Furthermore, there was no differences in DTI indices when comparing migraine directly with TTH. However, the lack of an association between WM microstructure and headache type must be viewed in the light of effect sizes and sample sizes. Most of the comparisons involving migraine and TTH actually had larger Cohen’s d values than many of the comparisons with significant findings but included fewer individuals. Hence, despite its size, this study was probably underpowered to detect if type of headache was associated with differences in WM microstructure.

It is not known why some studies report migraineurs to have higher WM diffusion compared to headache free individuals [11–13] whereas others report the opposite [14–17]. One explanation may be differences in basic participant characteristics due to differences in the mode of recruitment. In this regard the present authors believe it is of great importance that this study was based on the general population with no group differences in cardiovascular risk factors or socioeconomic status. Differences in demographical and clinical variables were also corrected for. Furthermore, the present migraine group was larger than the ones in previous studies which further strengthens the confidence in the present results.

The analyses on tract-average DTI indices confirmed the TBSS results of widespread WM microstructural differences between the headache free and those suffering from headache, more precisely TTH and/or new onset headache. The difference in tract-average values were almost completely explained by WMH, HADS, chronic pain and consumption of alcohol and over-the-counter painkillers. Comparisons of the tract volumes showed that, compared to the headache free, those with TTH had lower volume of CC and those with new onset headache had lower volume of CC and IFOF. This could not be explained by any of the covariates. It is important to note that a large number of analyses were performed and that the automated tractography analyses were not corrected for multiple comparisons. Hence, any significant findings should be interpreted with caution.

There are several strengths of the present study. First, compared to previous studies it was large with more power to detect group differences and less vulnerable to random errors. Second, the participants were randomly drawn among individuals attending a large longitudinal epidemiological study (HUNT) minimizing selection bias of clinic-based studies. Third, differences in diffusivity indices were investigated with regard to type of headache, frequency of headache attacks and evolution of headache. Fourth, all scans were performed on the same scanner. Fifth, both TBSS and automated tractography were applied in the analyses of the DTI data. The present study also has some limitations. First, there was a relatively long time interval from the participants answered the headache questionnaire (2006–2008 in HUNT 3) to when they were scanned (2007–2009). Morphological changes of the brain have been reported to both arise and recede within a year, although this has not been shown for DTI [10, 43]. Furthermore, it seems unlikely that the headache had improved dramatically in the majority during the time between the HUNT3 questionnaire and the MRI scanning (mean 1.2 years). Second, headache status was estimated using a questionnaire which is inferior to a clinical interview. However, the headache diagnoses in HUNT3 were validated showing acceptable accuracy [26]. The migraine diagnosis was highly specific but had lower sensitivity whereas this was opposite for the TTH diagnosis. Some true migraineurs were therefore probably incorrectly classified with TTH, which potentially diminished the differences between these groups. The headache categories regarding frequency of attacks and evolution of headache were not validated. Hence, caution must be taken when interpreting these specific analyses. Third, the evolution of the participant’s headache was based on data from only two time points with no information on headache history in between, which further warrants caution regarding these analyses. Fourth, we had no information on use of prophylactic medication. Fifth, we had no information on whether participants were scanned during or between attacks. Sixth, overadjustment may have occurred in some of the statistical models, as for instance headache and chronic pain are associated with each other and may have common causes linking them to WM microstructure.

In light of the present findings, future studies should investigate WM morphology related to the age of headache sufferers and the age of onset of the headache. Furthermore, to ensure sufficient statistical power to detect potential small differences, studies should be based on large samples and samples from the general population should be preferred over samples from clinics to avoid selection bias. Future studies should also not restrict their analyses to FA and MD but also investigate AD and RD.

In conclusion the present study found widespread higher WM diffusion in those suffering from headache compared to headache free individuals in the general population. The largest effects were seen in those with headache developed in middle-age. Overall, the effects were small and there was no dose-response relationship between headache frequency and WM microstructure. WMH, HADS score, chronic pain and consumption of alcohol and over-the-counter painkillers largely explained the findings in those with migraine, TTH or persistent headache, but to a far lesser degree in those with previous or new onset headache.

## Additional files

Additional file 1: **Table S1.** Scan parameters for the T1, T2 and FLAIR sequences. (DOCX 13 kb)

Additional file 2: **Table S2.** Intracranial abnormalities related to headache status. (DOCX 14 kb)

Additional file 3: **Table S3.** Rare intracranial abnormalities related to headache status. (DOCX 13 kb)

Additional file 4: **Table S4.** Number of individuals in each Fazeka’s grade in the different headache categories. (DOCX 15 kb)

Additional file 5: **Table S5.** Frequency of headache attacks in HUNT3 in the different headache categories. (DOCX 14 kb)

Additional file 6: **Table S6.** Diffusivity indices (mean values with standard deviations in parenthesis) of white matter tracts with significant differences between various headache groups obtained via automated tractography. Only significant comparisons are shown. (DOCX 16 kb)

Additional file 7: **Figure S1.** Differences in white matter FA, MD, AD and RD in the TBSS analyses corrected for age, sex, HADS, chronic pain and consumption of alcohol and over-the-counter painkillers. Significance level was *P* < 0.05 (two-tailed) and corrected for multiple comparisons with Threshold Free Cluster Enhancement and Family-Wise Error rate as implemented in Randomise. To improve visualization, the group differences were “thickened” using the tbss_fill script in FSL. The FSL 1 mm mean FA template was used as background image. (PNG 2254 kb)

Additional file 8: **Figure S2.** Differences in white matter FA, MD, AD and RD in the TBSS analyses corrected for age, sex, WMH, HADS, chronic pain and consumption of alcohol and over-the-counter painkillers. Significance level was *P* < 0.05 (two-tailed) and corrected for multiple comparisons with Threshold Free Cluster Enhancement and Family-Wise Error rate as implemented in Randomise. To improve visualization, the group differences were “thickened” using the tbss_fill script in FSL. The FSL 1 mm mean FA template was used as background image. (PNG 1722 kb)

## Data Availability

The present data are the property of HUNT research centre and can only be accessed through direct contact with the research centre.
