# Supplementary material for: Diffusion tensor imaging in middle-aged headache sufferers in the general population: a cross-sectional population-based imaging study in the Nord-Trøndelag health study (HUNT-MRI)
Source: J Headache Pain. 2019 Jul 10;20(1):78. doi: 10.1186/s10194-019-1028-6 (PMC6734377; doi:10.1186/s10194-019-1028-6)
Supplement: Supplementary file 1 — Table S1. Scan parameters for the T1, T2 and FLAIR sequences. (DOCX 13 kb) [file 10194_2019_1028_MOESM1_ESM.docx]

**Supplementary table 1.** Scan parameters for the T1, T2 and FLAIR sequences

| **MRI sequence** | **Matrix size** | **NSA** | **TR**  **(ms)** | **TE**  **(ms)** | **Flip-angle** | **Slice thickness (mm)** | **Gap (mm)** | **Overlap (mm)** | **FOV**  **(mm)** |
| --- | --- | --- | --- | --- | --- | --- | --- | --- | --- |
| T1 W | 192x192 | 1 | 10.2 | 4.1 | 10 | 1.2 | 0 | 0 | 240 |
| T2 W | 512x320 | 2 | 7840.0 | 95.3 | 90 | 4.0 | 1 | 0 | 230 |
| FLAIR | 256x224 | 1 | 11,002.0 | 122.9 | 90 | 4.0 | 1 | 0 | 230 |

T1 W, T1 weighted; T2 W, T2 weighted; FLAIR, fluid attenuated inversion recovery; NSA, number of signal averages; TR, time of repetition; TE, time of echo; FOV, field of view
