# Supplementary material for: Diffusion tensor imaging in middle-aged headache sufferers in the general population: a cross-sectional population-based imaging study in the Nord-Trøndelag health study (HUNT-MRI)
Source: J Headache Pain. 2019 Jul 10;20(1):78. doi: 10.1186/s10194-019-1028-6 (PMC6734377; doi:10.1186/s10194-019-1028-6)
Supplement: Supplementary file 2 — Table S2. Intracranial abnormalities related to headache status. (DOCX 14 kb) [file 10194_2019_1028_MOESM2_ESM.docx]

**Supplementary table 2.** Intracranial abnormalities related to headache status.

|  | Total | Any headache in HUNT3 | Migraine in HUNT3 | TTH in HUNT3 | Previous headache | New onset headache | Persistent headache | Headache free |
| --- | --- | --- | --- | --- | --- | --- | --- | --- |
| **Intracranial abnormality** | **n=810** | **n=246** | **n=69** | **n=76** | **n=117** | **n=49** | **n=178** | **n=277** |
| Cerebral infarctions (%) | 14 (1.7) | 3 (1.2) | 1 (1.4) | 0 (0.0) | 2 (1.7) | 0 (0.0) | 3 (1.7) | 7 (2.5) |
| Silent (%) | 13 (1.6) | 3 (1.2) | 1 (1.4) | 0 (0.0) | 2 (1.7) | 0 (0.0) | 3 (1.7) | 6 (2.2) |
| Clinical (%) | 1 (0.1) | 0 (0.0) | 0 (0.0) | 0 (0.0) | 0 (0.0) | 0 (0.0) | 0 (0.0) | 1 (0.4) |
| Cysts (%) | 33 (4.1) | 9 (3.7) | 2 (2.9) | 2 (2.6) | 9 (7.7) | 1 (2.0) | 8 (4.5) | 10 (3.6) |
| Arachnoid (%) | 20 (2.5) | 6 (2.4) | 1 (1.4) | 2 (2.6) | 4 (3.4) | 0 (0.0) | 6 (3.4) | 6 (2.2) |
| Non-arachnoid (%) | 13 (1.6) | 3 (1.2) | 1 (1.4) | 0 (0.0) | 5 (4.3) | 1 (2.0) | 2 (1.1) | 4 (1.4) |
| Aneurysm (%) | 15 (1.9) | 4 (1.6) | 0 (0.0) | 1 (1.3) | 2 (1.7) | 3 (6.1) | 1 (0.6) | 3 (1.1) |
| Microhaemorrhage (%) | 11 (1.4) | 4 (1.6) | 0 (0.0) | 2 (2.6) | 2 (1.7) | 1 (2.0) | 3 (1.7) | 4 1.4) |
| Rare abnormalities (%) | 18 (2.2) | 11 (4.5) | 2 (2.9) | 3 (3.9) | 4 (3.4) | 2 (4.1) | 9 (5.1) | 3 (1.1) |
