# Supplementary material for: Diffusion tensor imaging in middle-aged headache sufferers in the general population: a cross-sectional population-based imaging study in the Nord-Trøndelag health study (HUNT-MRI)
Source: J Headache Pain. 2019 Jul 10;20(1):78. doi: 10.1186/s10194-019-1028-6 (PMC6734377; doi:10.1186/s10194-019-1028-6)
Supplement: Supplementary file 3 — Table S3. Rare intracranial abnormalities related to headache status. (DOCX 13 kb) [file 10194_2019_1028_MOESM3_ESM.docx]

**Supplementary table 3.** Rare intracranial abnormalities related to headache status.

| **Rare abnormality** | **N** | **No. of lesions per headache category** |
| --- | --- | --- |
| Occlusion of the internal carotid artery | 3 | 1 with previous headache and 2 with persistent headache |
| Pituitary tumour | 3 | 1 with previous headache, 1 with new onset headache and 1 with persistent headache |
| Chiari malformation | 2 | 1 with persistent headache |
| Cavernous haemangioma | 2 | 1 with new and 1 with persistent headache |
| Calcification of parenchyma | 2 | 1 with previous headache and 1 headache free |
| Vestibular schwannoma | 1 | 1 with persistent headache |
| Meningioma | 1 | 1 with previous headache |
| Carotid siphon stenosis | 1 | 1 with persistent headache |
| Venous angioma | 1 | 1 headache free |
| Mega cisterna magna | 1 | 1 with persistent headache |
| Postoperative alterations | 1 | 1 headache free |

None of the participants had cerebral contusion, AV-malformation, malignant neoplasm, stenosis in medial cerebral artery, grey matter heterotopia, progressive supranuclear palsy or multiple sclerosis.
