# Supplementary material for: Diffusion tensor imaging in middle-aged headache sufferers in the general population: a cross-sectional population-based imaging study in the Nord-Trøndelag health study (HUNT-MRI)
Source: J Headache Pain. 2019 Jul 10;20(1):78. doi: 10.1186/s10194-019-1028-6 (PMC6734377; doi:10.1186/s10194-019-1028-6)
Supplement: Supplementary file 4 — Table S4. Number of individuals in each Fazeka’s grade in the different headache categories. (DOCX 15 kb) [file 10194_2019_1028_MOESM4_ESM.docx]

**Supplementary table 4.** Number of individuals in each Fazeka’s grade in the different headache categories

|  | **Fazeka’s score** | | | | **Total** |
| --- | --- | --- | --- | --- | --- |
|  | **Grade 0** | **Grade 1** | **Grade 2** | **Grade 3** |  |
| Headache free | 160 (57.8%) | 97 (35.0%) | 18 (6.5%) | 2 (0.7%) | 277 (100.0%) |
| Any headache in HUNT3 | 128 (52.0%) | 92 (37.4%) | 19 (7.7%) | 7 (2.8%) | 246 (100.0%) |
| Migraine in HUNT3 | 39 (56.5%) | 23 (33.3%) | 5 (7.2%) | 2 (2.9%) | 69 (100.0%) |
| TTH in HUNT3 | 35 (46.1%) | 31 (40.8%) | 9 (11.8%) | 1 (1.3%) | 76 (100.0%) |
| Previous headache | 66 (56.4%) | 42 (35.9%) | 9 (7.7%) | 0 (0.0%) | 117 (100.0%) |
| Persistent headache | 98 (55.1%) | 62 (34.8%) | 12 (6.7%) | 6 (3.4%) | 178 (100.0%) |
| New onset headache | 23 (46.9%) | 20 (40.8%) | 6 (12.2%) | 0 (0.0%) | 49 (100.0%) |
|  |  |  |  |  |  |
| Headache <1 day/month | 40 (58.0%) | 23 (33.3%) | 4 (5.8%) | 2 (2.9%) | 69 (100.0%) |
| Headache 1-6 days/month | 64 (47.4%) | 55 (40.7%) | 12 (8.9%) | 4 (3.0%) | 135 (100.0%) |
| Headache 7-14 days/month | 17 (60.7%) | 8 (28.6%) | 2 (7.1%) | 1 (3.6%) | 28 (100.0%) |
| Headache >14 days/month | 7 (53.8%) | 5 (38.5%) | 1 (7.7%) | 0 (0.0%) | 13 (100.0%) |

There was no significant correlation between Fazeka’s score and headache attack frequency (*X*2 (9, N=245)=3.73, p=0.9).
