# Supplementary material for: Diffusion tensor imaging in middle-aged headache sufferers in the general population: a cross-sectional population-based imaging study in the Nord-Trøndelag health study (HUNT-MRI)
Source: J Headache Pain. 2019 Jul 10;20(1):78. doi: 10.1186/s10194-019-1028-6 (PMC6734377; doi:10.1186/s10194-019-1028-6)
Supplement: Supplementary file 5 — Table S5. Frequency of headache attacks in HUNT3 in the different headache categories. (DOCX 14 kb) [file 10194_2019_1028_MOESM5_ESM.docx]

**Supplementary table 5.** Frequency of headache attacks in HUNT3 in the different headache categories.

|  | Any headache in HUNT3 | Migraine in HUNT3 | TTH in HUNT3 | New onset headache | Persistent headache |
| --- | --- | --- | --- | --- | --- |
|  | **n=245*** | **n=68*** | **n=76** | **n=49** | **n=177*** |
| Headache <1 day/month | 69  (28.2%) | 19  (27.9%) | 0  (0.0%) | 20  (40.8%) | 49  (27.7%) |
| Headache 1-6 days/month | 135  (55.1%) | 38  (55.9%) | 62  (81.6%) | 24  (49.0%) | 94  (53.1%) |
| Headache 7-14 days/month | 28  (11.4%) | 11  (16.2%) | 11  (14.5%) | 4  (8.2%) | 23  (13.0%) |
| Headache >14 days/month | 13  (5.3%) | 0  (0.0%) | 3  (3.9%) | 1  (2.0%) | 11  (6.2%) |

*One participant with migraine in both HUNT2 and HUNT3 had missing data on headache attack frequency
Previous headache = Not applicable (since they were headache free in HUNT3)
Headache free = Not applicable (since they were headache free in HUNT3)
