# Supplementary material for: Diffusion tensor imaging in middle-aged headache sufferers in the general population: a cross-sectional population-based imaging study in the Nord-Trøndelag health study (HUNT-MRI)
Source: J Headache Pain. 2019 Jul 10;20(1):78. doi: 10.1186/s10194-019-1028-6 (PMC6734377; doi:10.1186/s10194-019-1028-6)
Supplement: Supplementary file 6 — Table S6. Diffusivity indices (mean values with standard deviations in parenthesis) of white matter tracts with significant differences between various headache groups obtained via automated tractography. Only significant comparisons are shown. (DOCX 16 kb) [file 10194_2019_1028_MOESM6_ESM.docx]

**Supplementary table 4.** Diffusivity indices (mean values with standard deviations in parenthesis) of white matter tracts with significant differences between various headache groups obtained via automated tractography. Only significant comparisons are shown.

| **White matter tract** | **Diffusivity index** | **Headache groups compared** | | **Cohen’s d** | **P-values** |
| --- | --- | --- | --- | --- | --- |
|  |  | **Headache free** | **Any headache in HUNT3** |  |  |
| ILF | MD | 0.844 (0.030) | 0.845 (0.032) | 0.03 | 0.038* |
|  | AD | 1.159 (0.035) | 1.162 (0.037) | 0.08 | 0.013* |
|  | AD | 1.159 (0.035) | 1.162 (0.037) | 0.08 | 0.042† |
|  |  | **Headache free** | **Migraine in HUNT3** |  |  |
| CST | FA | 0.393 (0.011) | 0.389 (0.013) | 0.33 | 0.047‡ |
|  |  | **Headache free** | **TTH in HUNT3** |  |  |
| IFOF | MD | 0.826 (0.028) | 0.831 (0.028) | 0.18 | 0.026* |
|  | AD | 1.126 (0.033) | 1.134 (0.034) | 0.24 | 0.018* |
|  | RD | 0.676 (0.026) | 0.680 (0.027) | 0.15 | 0.042* |
| ILF | MD | 0.844 (0.030) | 0.849 (0.033) | 0.16 | 0.019* |
|  | AD | 1.159 (0.035) | 1.167 (0.037) | 0.22 | 0.010* |
|  | AD | 1.159 (0.035) | 1.167 (0.037) | 0.22 | 0.035† |
|  | AD | 1.158 (0.035) | 1.167 (0.037) | 0.25 | 0.048‡ |
|  | RD | 0.686 (0.030) | 0.690 (0.033) | 0.13 | 0.037* |
| CST | AD | 1.151 (0.030) | 1.157 (0.034) | 0.19 | 0.028* |
|  |  | **Headache free** | **Previous headache** |  |  |
| CST | FA | 0.393 (0.011) | 0.390 (0.012) | 0.26 | 0.044‡ |
|  | FA | 0.393 (0.011) | 0.390 (0.012) | 0.26 | 0.042# |
|  | AD | 1.151 (0.030) | 1.156 (0.031) | 0.16 | 0.049* |
|  |  | **Headache free** | **New onset headache** |  |  |
| IFOF | MD | 0.826 (0.028) | 0.833 (0.030) | 0.24 | 0.048* |
|  | AD | 1.126 (0.033) | 1.138 (0.036) | 0.35 | 0.011* |
|  | AD | 1.126 (0.033) | 1.138 (0.036) | 0.35 | 0.026† |
| ILF | MD | 0.844 (0.030) | 0.853 (0.028) | 0.31 | 0.012* |
|  | MD | 0.844 (0.030) | 0.853 (0.028) | 0.31 | 0.029† |
|  | AD | 1.159 (0.035) | 1.172 (0.034) | 0.38 | 0.005* |
|  | AD | 1.159 (0.035) | 1.172 (0.034) | 0.38 | 0.011† |
|  | AD | 1.158 (0.035) | 1.172 (0.034) | 0.41 | 0.040‡ |
|  | RD | 0.686 (0.030) | 0.694 (0.029) | 0.27 | 0.030* |
| SLF | AD | 1.079 (0.030) | 1.086 (0.036) | 0.21 | 0.041* |
|  |  | **Headache free** | **Persistent headache** |  |  |
| CING | FA | 0.324 (0.013) | 0.326 (0.012) | 0.16 | 0.032* |
|  | FA | 0.324 (0.013) | 0.326 (0.012) | 0.16 | 0.023† |
|  | RD | 0.711 (0.031) | 0.701 (0.032) | 0.32 | 0.038† |

AD=axonal diffusion; FA=fractional anisotropy; MD=mean diffusivity; RD=radial diffusivity
MD, AD and RD are given in 10^-3^ mm^2^/sec
Between-group differences were investigated with ANCOVA thresholded at *P*<0.05 (two-tailed).

*Corrected for age and sex
†Corrected for age, sex and WMH
‡ Corrected for age, sex, ICV, HADS, chronic pain and consumption of alcohol and over-the-counter painkillers
# Corrected for age, sex, WMH, ICV, HADS, chronic pain and consumption of alcohol and over-the-counter painkillers
