# Supplementary figures and images for: Diffusion tensor imaging in middle-aged headache sufferers in the general population: a cross-sectional population-based imaging study in the Nord-Trøndelag health study (HUNT-MRI)
Source: J Headache Pain. 2019 Jul 10;20(1):78. doi: 10.1186/s10194-019-1028-6 (PMC6734377; doi:10.1186/s10194-019-1028-6)

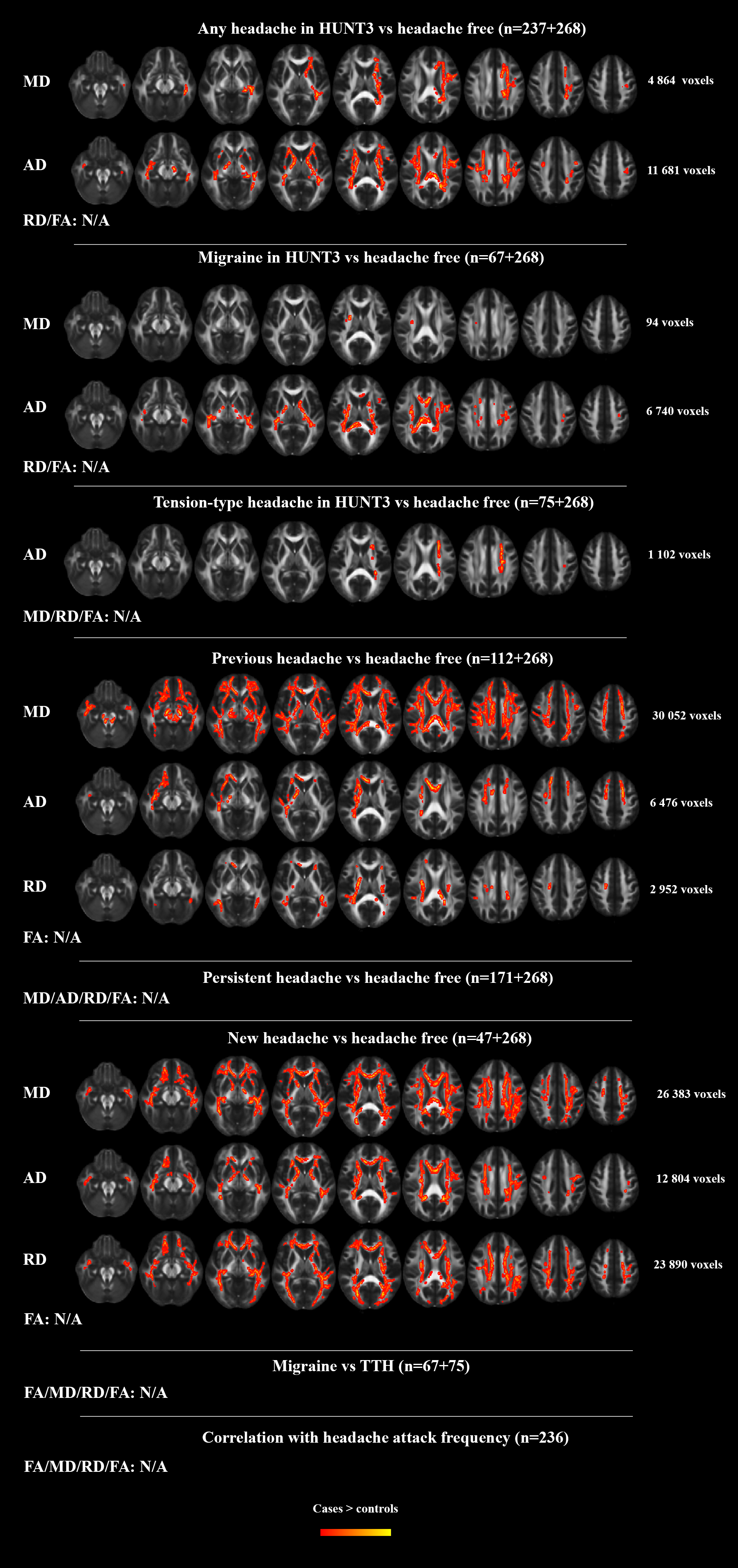

Supplement: Supplementary file 7 — Figure S1. Differences in white matter FA, MD, AD and RD in the TBSS analyses corrected for age, sex, HADS, chronic pain and consumption of alcohol and over-the-counter painkillers. Significance level was P < 0.05 (two-tailed) and corrected for multiple comparisons with Threshold Free Cluster Enhancement and Family-Wise Error rate as implemented in Randomise. To improve visualization, the group differences were “thickened” using the tbss_fill script in FSL. The FSL 1 mm mean FA template was used as background image. (PNG 2254 kb) [file 10194_2019_1028_MOESM7_ESM.png]

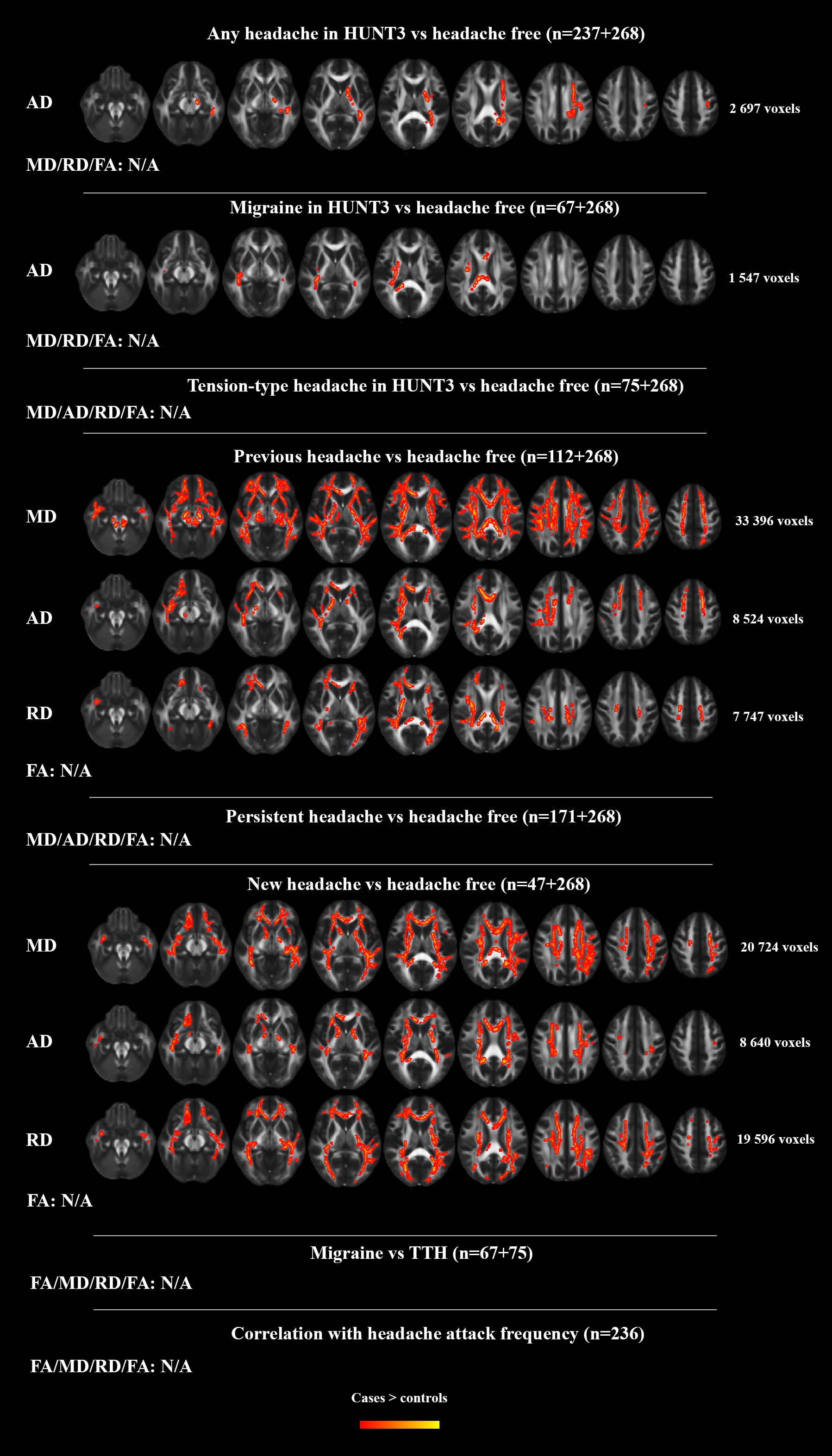

Supplement: Supplementary file 8 — Figure S2. Differences in white matter FA, MD, AD and RD in the TBSS analyses corrected for age, sex, WMH, HADS, chronic pain and consumption of alcohol and over-the-counter painkillers. Significance level was P < 0.05 (two-tailed) and corrected for multiple comparisons with Threshold Free Cluster Enhancement and Family-Wise Error rate as implemented in Randomise. To improve visualization, the group differences were “thickened” using the tbss_fill script in FSL. The FSL 1 mm mean FA template was used as background image. (PNG 1722 kb) [file 10194_2019_1028_MOESM8_ESM.png]
